# Temporal Processing and Speech Perception in Noise by Listeners with Auditory Neuropathy

**DOI:** 10.1371/journal.pone.0055995

**Published:** 2013-02-07

**Authors:** Vijaya Kumar Narne

**Affiliations:** All India Institute of Speech and Hearing, Mysore, India; Baycrest Hospital, Canada

## Abstract

**Aim:**

The present study evaluated the relation between speech perception in the presence of background noise and temporal processing ability in listeners with Auditory Neuropathy (AN).

**Method:**

The study included two experiments. In the first experiment, temporal resolution of listeners with normal hearing and those with AN was evaluated using measures of temporal modulation transfer function and frequency modulation detection at modulation rates of 2 and 10 Hz. In the second experiment, speech perception in quiet and noise was evaluated at three signal to noise ratios (SNR) (0, 5, and 10 dB).

**Results:**

Results demonstrated that listeners with AN performed significantly poorer than normal hearing listeners in both amplitude modulation and frequency modulation detection, indicating significant impairment in extracting envelope as well as fine structure cues from the signal. Furthermore, there was significant correlation seen between measures of temporal resolution and speech perception in noise.

**Conclusion:**

Results suggested that an impaired ability to efficiently process envelope and fine structure cues of the speech signal may be the cause of the extreme difficulties faced during speech perception in noise by listeners with AN.

## Introduction

Auditory Neuropathy (AN) is a term used to describe auditory disorders with dysfunction of the auditory nerve in the presence of preserved cochlear outer hair-cell function [1], [2]. One main characteristic of AN is disrupted auditory nerve activity, evidenced by absent or severely abnormal auditory brainstem response, with normal or near normal cochlear outer hair cell function, as observed by the presence of oto-acoustic emissions and/or cochlear microphonics.

Psychoacoustic experiments conducted on listeners with AN indicate a significant impairment in temporal processing and this leads to extreme difficulty in understanding speech [3], [4]. Speech perception difficulties (in quiet and background noise) are not unique to listeners with AN. Listeners with cochlear hearing loss also have difficulty in understanding speech in quiet, more so in the presence of competing signals [5]. In listeners with AN, speech perception difficulties in quiet range from minimal to severe and these difficulties are exaggerated in presence of competing background noise [3], [4], [6]. Studies have shown that these perceptual difficulties cannot be explained based on the degree of hearing loss or audibility [7], [8]. Attempts have been made through psychoacoustic and simulation studies to explain factors responsible for perceptual difficulties experienced by listeners with AN in quiet [8]–[10]. These investigators have suggested that perceptual difficulties experienced by listeners with AN may be due to an impaired ability to follow amplitude variations in speech signals (temporal envelope cues). Further, it has been hypothesized that this impaired ability to follow amplitude variations may reduce the consonant-vowel distinction, resulting in extreme difficulty in understanding speech [9].

Narne and Vanaja [6] have evaluated speech identification scores in quiet and in the presence of noise (signal to noise ratio of 10, 5 and 0 dB). A greater reduction in identification scores in noise was observed for listeners with AN than those with normal hearing. Also, identification scores in noise were much lower for those who had poor identification scores (<50%), than those who had good identification scores in quiet (>50%). Similar results have been demonstrated for children by Rance et al. [11] and for adults by Zeng and Liu [12]. The exact mechanisms underlying these extreme perceptual difficulties in noise are not clear.

A number of previous investigators have examined reasons for the reduction in speech identification scores in the presence of background noise for listeners with normal hearing [13]–[15]. Background noise reduces the modulation depth of temporal envelopes and introduces spurious modulations, which obscures the relevant speech modulations [15].This has been proposed as one of the major reasons for reduced speech intelligibility in the presence of competing signals [13], [14], [16], [17]. From the aforementioned studies one can hypothesize that impaired ability to follow amplitude variations in speech signal may in part be the root cause of the extreme difficulty experienced by listeners with AN in understanding speech in the presence of noise. But this assumption doesn't hold good in the case of those individuals with AN, who have poorer speech identification scores in the presence of noise but have good amplitude modulation detection ability and speech identification scores in quiet within normal limits.

Studies manipulating the temporal fine structure (TFS) information using vocoder techniques have shown that TFS information is important for speech perception in both steady state and modulating noises [18], [19]. Investigations have been carried out to study the processing of TFS in listeners with cochlear hearing loss using psycho-acoustical [20]–[22] and speech perception experiments [19], [23]–[25]. Results of these studies indicate that impaired processing of TFS may account partly for the poor performance presented by persons with cochlear hearing loss in competing noise.

The aforesaid studies dealing with temporal envelope and TFS hint towards a hypothesis that one of the possible reasons for extreme difficulty in understanding speech in the presence of noise by listeners with AN may be associated with impaired processing of both temporal envelope and TFS cues. Previous investigators have assessed temporal envelope processing ability using temporal modulation transfer function (TMTF) in listeners with AN. Results of these studies have demonstrated impaired ability of listeners with AN in detecting both slow and fast temporal modulations, with more impairment in processing faster modulations than slower modulations [3], [4], [26]. Further, a good correlation has been reported between the TMTF threshold and speech perception scores in quiet for listeners with AN [3], [4], [26]. TFS processing in listeners with AN has been assessed only by two investigators. First, Rance et al. [3] have assessed TFS information processing using frequency modulation discrimination in children with AN and they showed that TFS processing was significantly impaired in children with AN. Zeng et al. [10] have assessed lateralization for pure-tone using phase cues and in detecting binaural beats in listeners with AN. Results showed that listeners with AN were impaired to use phase cues and also in detecting beats. These findings are consistent with impaired processing of temporal fine structure information. However, currently there are no studies that have investigated the relationship between speech perception scores in noise, temporal envelope and TFS processing in listeners with AN. Hence, the present study aims to investigate the relation between speech perception in noise and performance on psycho-acoustical tasks that are thought to depend on the temporal processing in listeners with AN.

To address this aim, two experiments were conducted. The first experiment assessed temporal envelope processing using auditory temporal modulation transfer function (TMTF) [27] and fine structure processing using frequency modulation discrimination [28]. In the second experiment, speech perception scores were assessed in quiet and in three SNR conditions.

## Methods

### Listeners

#### Auditory Neuropathy (AN)

25 listeners with AN (13 males and 12 females) in the age range of 12 to 26 years with a mean age of 18 years participated in the study. The listeners were diagnosed based on observable otoacoustic emissions, absent acoustic reflexes and absent ABRs. All listeners were native speakers of Kannada (a Dravidian language spoken in a southern state of India). The pure-tone average (mean of 0.5, 1, 2 and 4 kHz) ranged from 11 to 53 dB HL with an average of 37 dB HL for the right ear and 36 dB HL for the left ear. Twenty listeners had low-frequency hearing loss (rising configuration) and five had flat hearing loss. Table 1 provides the demographic and audiological details of the listeners with AN.

**Table 1:** Audiological profile of participants with auditory neuropathy.

S. No	Age (yr)	Duration (yr)	Sex	Pure-tone Average (dB HL)		Speech Identification scores in (%)
				Right	Left	
AN1	20	10.00	Male	31	34	72
AN2	20	5.00	Female	43	39	48
AN3	23	12.00	Female	32	35	84
AN4*	22	15.00	Male	37	42	100
AN5*	18	6.00	Male	19	10	100
AN6	22	10.00	Female	53	44	8
AN7	14	4.00	Male	33	28	16
AN8	23	7.00	Male	29	33	60
AN9	26	16.00	Female	33	29	52
AN10	15	2.00	Male	50	41	20
AN11	24	12.00	Male	43	51	0
AN12	19	6.00	Female	41	47	72
AN13	15	3.00	Male	29	33	20
AN14	18	5.00	Female	35	42	96
AN15	15	3.00	Male	17	13	40
AN16*	24	14.00	Female	16	11	100
AN17	23	12.00	Male	26	30	0
AN18	12	2.00	Female	42	47	8
AN19	15	3.00	Male	51	52	48
AN20	15	3.00	Female	49	47	44
AN21*	19	3.00	Male	35	34	100
AN22	15	2.00	Female	34	31	84
AN23	12	2.00	Male	53.3	53.3	8
AN24	19	5.00	Female	32	35	84
AN25	18	5.00	Female	48	44	0

All participants had absent ABRs, acoustic reflexes, and present OAEs.

* Indicates the four exceptional participants who had normal speech identification scores in quietand Normal TMTF thresholds.

#### Normal Hearing

This group consisted of twenty five listeners (12 male and 13 female) with normal hearing, in the age range of 15 to 30 years with a mean age of 23 years. The primary language of all listeners was Kannada. It was ascertained from a structured interview that none of these listeners had any difficulty in understanding speech in daily listening conditions, and that they did not have any history of neurologic or otologic disorder. All the listeners had pure-tone thresholds of less than 15 dB HL [39] at octave frequencies between 0.25 to 8 kHz and a speech identification score of greater than 90% at 40 dB SL (ref: pure-tone average at 0.5, 0.1, 0.2 and 4 kHz). Immittance evaluation and recording of auditory brainstem responses as well as transient otoacoustic emissions revealed normal findings in all the listeners.

### Ethical Considerations

In the present study, all the testing procedures were approved by institutional review board (All India Institute of Speech and Hearing). The procedures involved in the present study were non-invasive and all the procedures were explained to the patients and their family members before testing and written informed consent was taken from all the patients for adults and from the family members for minors for participating in the study.

### Experiment-1: Temporal processing

#### Stimuli

a. Temporal Modulation Transfer Function (TMTF).

Two stimuli, unmodulated white noise and sinusoidally amplitude modulated white noise, of 500 ms duration with raised-cosine ramp of 20 ms were used. The stimuli were generated using a 16-bit digital to analog converter with a sampling frequency of 44.1 kHz and were low pass filtered with a cut off frequency of 20 kHz. The modulated signal was derived by multiplying the white noise by a dc-shifted sine wave. The depth of the modulation was controlled by varying the amplitude of the modulating sine wave. Equation (1) gives the expression describing the sinusoidally amplitude modulated stimuli.
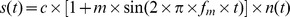


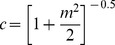
Where m is the modulation depth (0<m<1), *fm* is the modulation frequency in Hz (2, 4, 8, 16, 32, 64, 128, 256, 512), and n(t) is the waveform of the white noise. The term c is a multiplicative compensation term [40] set such that the overall power was same for modulated and unmodulated stimuli. The level of presentation was randomized over a range of 10 dB with mean level of presentation being approximately 80 dB SPL. The level was varied over 10 dB to avoid the intensity cues.

b. Frequency Modulation Detection.

Stimuli were computed in the time domain according to the Equation (2):
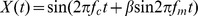


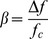
Where *fc* is the carrier frequency (0.5, 1, 2&4 kHz), *f*_m_ is the modulation rate (2&10 Hz), and Δ*f* is the modulation depth. On each trial, three successive stimuli were presented, one frequency modulated and the other two unmodulated. The order of the three stimuli in each pair was randomized. Each stimulus had an overall duration of 500 ms, including raised-cosine rise/fall times of 20 ms. The time interval between the stimuli was 500 ms.

#### Procedure

Thresholds were estimated based on a 3 AFC procedure with a 2-down 1-up tracking method, estimating the 70.7% correct point on psychometric function [41]. In this procedure, the target signal (amplitude and frequency modulation) was reduced after 2 correct responses, and target signal was increased after 1 in-correct response. In the above two tasks, stimuli were presented at a comfortable level (approximately 80 dB SPL). The stimuli were played from a computer and routed through an audiometer (Madsen OB-922). The listeners received the signal from the loudspeaker(C 115 Martin Audio), kept at a distance of one meter at 0° azimuth.

a. Temporal Modulation Transfer Function (TMTF)

In the TMTF experiment, the participant's task was to identify the interval containing the amplitude modulation. No feedback was given. The step size and amplitude modulation thresholds were based on the modulation depth in decibels (20×log_10_ (m)). The step size was initially 4 dB and was reduced to 2 dB after two reversals. The mean of the levels at the last eight reversals in a block of 14 was taken as threshold. The worst threshold that could be measured was 0 dB, and it corresponded to a modulation depth of one (100% modulated noise). While estimating the TMTF threshold, it was noticed that many listeners could not detect even 100% at some modulation frequencies. The procedure was terminated at that level and the data of those frequencies were not considered for further analysis.

b. Frequency Modulation Detection

In the frequency modulation paradigm, the participant's task was to detect the interval containing the frequency modulated tone. The modulation depth was some value above 0 and represents a range over which the FM tone varied in frequency. The amount of FM was changed by a factor of 1.5 until four reversals had occurred, and by a factor of 1.25 for the subsequent eight reversals. The threshold was estimated as the geometric mean of the amounts of FM at the last eight reversals.

### Experiment 2: Speech Identification

#### Stimuli

The speech stimulus was a set of bi-syllabic wordlists in Kannada, developed by Yathiraj and Vijaylakshami [42]. It consisted of four lists, each with 25 bi-syllabic words, which were phonetically balanced and were equally difficult. The words were spoken in conversational style by a female native speaker of Kannada. They were digitally recorded in an acoustically treated room, on a data acquisition system using a 44.1 kHz sampling frequency and 32-bit analog to digital converter.

In the experiments involving background noise, each word was mixed with speech spectrum shaped noise at SNRs of 0, 5 and 10 dB. The speech spectrum shaped noise was generated by randomizing the phase of Fourier spectrum of concatenated words of original signals of all the four lists.

#### Procedure

The listeners listened to speech tokens individually in a double-walled, acoustically treated room where the ambient noise levels were within permissible limits [43]. The speech stimuli were played from a PC, with a sound card of high definition Realtek, at 44.1 kHz sampling rate and routed to a calibrated [44] diagnostic audiometer (Madson OB-922 with speaker). The listeners received the signal from the loudspeaker (C 115 Martin Audio) of the audiometer kept at a distance of one meter at0° azimuth. Speech stimuli were presented at 40 dB SL (with reference to PTA). No practice was given for listeners. None of the lists were repeated for any of the listeners, as there were four lists and four conditions. The order of presentation of conditions was randomized across the listeners. Listeners had to repeat the speech token heard. The speech recognition scores were calculated by counting the number of words correctly repeated.

## Results

### Temporal Modulation Transfer Function

**Figure 1:**
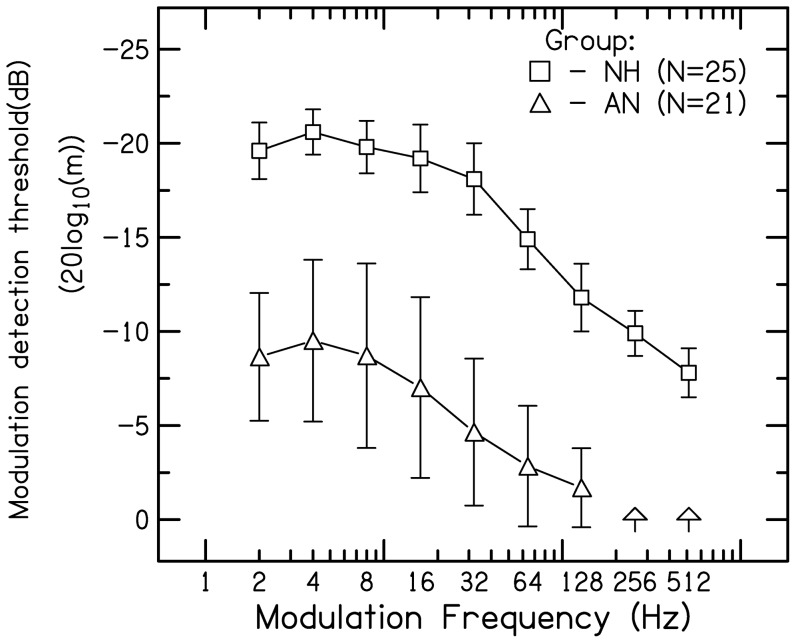
Amplitudemodulation detection thresholds as a function of modulation frequency in listeners with normal hearing (NH) and those with auditory neuropathy (AN). The opensquarefornormal hearing, open trianglefor listeners with AN. The open trianglewith downward arrow indicates thresholds >0 dB.

Figure 1 shows the mean TMTF, with error bars representing the standard deviation (SD) for listeners with AN and those with normal hearing. Four listeners with AN (#AN4, #AN5, #AN16, #AN21) showed normal performance in TMTF and speech identification scores in quiet. Data of these subjects were not included in the further analysis and they are discussed separately in the discussion section. As shown in Figure 1, for listeners with normal hearing, the average amplitude modulation detection threshold for low modulation frequencies (*fm* = 2–16 Hz) was −21 dB. Whereas for listeners with AN, thresholds were approximately 12 dB higher than those for listeners with normal hearing, i.e., the average amplitude modulation detection threshold was around −9.7 dB. A majority of the listeners with AN (20listeners) could not detect a modulation depth of 0 dB when the modulation frequency was 128 Hz or higher. The data at these modulation frequencies (128 Hz, 256 and 512 Hz) were not included for further analysis.

A mixed-model ANOVA (for repeated measures), with modulation frequency (6 levels) as a within-subject factor and group (2 levels) as a between-subject factor, was performed. This revealed a significant main effect of modulation frequency (F_(5, 2.76)_ = 378.5, p<0.01) and group (F_(1, 35.8)_ = 238.76, p<0.01). There was a significant interaction between group and modulation frequency (F_(5, 2.76)_ = 26.8, p<0.01), indicating that the difference in sensitivity, between the groups was not same for all the modulation frequencies. From Figure 1, it can be discerned that the difference between the two groups was smaller at low modulation frequencies and larger at higher modulation frequencies. Bonferroni's post hoc analysis for two-way interaction (modulation frequency×group) revealed a significant difference between normal hearing listeners and AN across all modulation frequencies. The TMTFs of both the groups resembled a low-pass filter. Each participant's TMTF was fitted with a first-order low-pass filter from which the peak sensitivity and 3 dB cutoff frequency measures were obtained. The fitted function is defined by Equation 3.
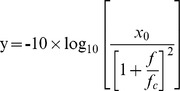
where y is the modulation index (m) in dB (−20 log_10_ m), *f* is the modulation frequency in Hz, −10 log (x_0_) is the peak sensitivity in dB and *fc* is the 3 dB cutoff frequency in Hz. The fitted function was adopted from Zeng et al. [10] and the curve fit program was implemented in MATLAB-7.9.

For listeners with normal hearing the average peak sensitivity was −21 dB (SD = 1.1) and fc was 67.5 Hz (SD = 10.5). For listeners with AN, the average peak sensitivity was −9.6 dB (SD = 4.5) and fc was 45.2 Hz (SD = 25.5). An independent samples ‘t’ test showed that difference in means was statistically significant for peak sensitivity (t = 15.6, p<0.01) but fc (t = −1.5, p<0.122) did not reach significance. The RMS error for the fitted function across the listeners ranged from 0.8 to 2.2 dB for listeners with normal hearing and from 0.8 to 3.4 dB for listeners with AN.

### Frequency Modulation Detection

**Figure 2:**
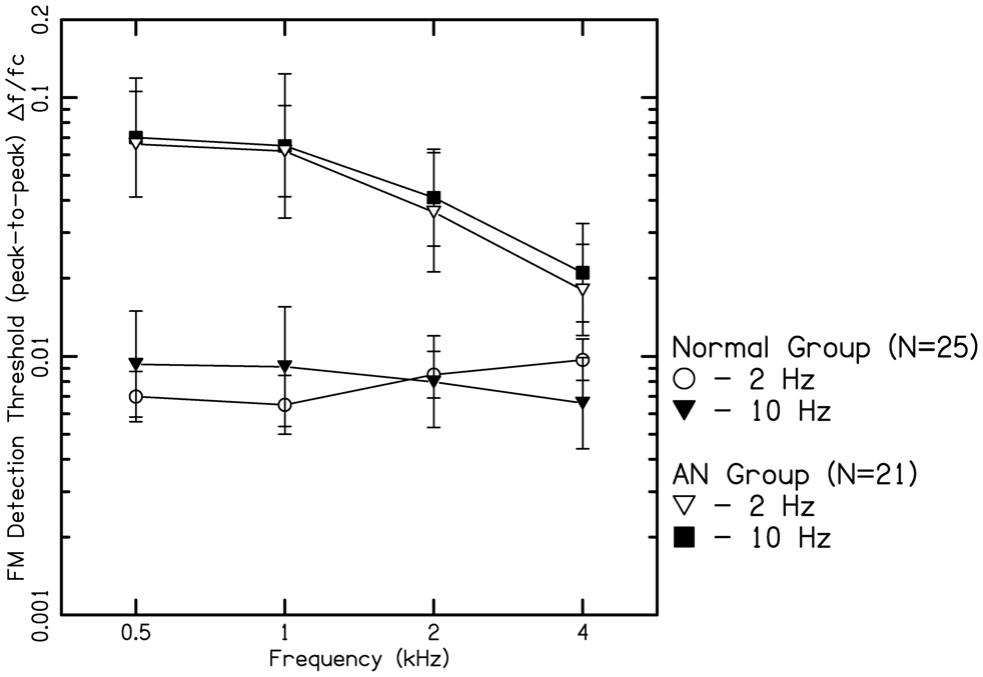
Frequency modulation detection threshold (FMDL) for 2 Hz and 10 Hz modulation frequencies as function of carrier frequencies (0.5, 1, 2, and 4 kHz) expressed as peak-to-peak deviation divided by center frequency in listeners with AN and normal hearing.

Figure 2 shows frequency modulation detection thresholds (FMDL) for modulation rates of 2 & 10 Hz for three carrier frequencies of 0.5, 1, 2&4 kHz. Thresholds have been plotted as the peak-to-peak frequency deviation divided by the center frequency. In normal hearing listeners, for FM at slow modulation rate (2 Hz), FMDLs were lower for low carrier frequencies (0.5 & 1 kHz) than higher carrier frequencies (2 & 4 k Hz). In contrast, FMDLs were lower for higher carrier frequencies (2 & 4 k Hz) than lower carrier frequency (0.5 & 1 kHz) for FM at high rate(10 Hz). Whereas, in listeners with AN, FMDLs were 10 times higher compared to normal hearing listeners at low carrier frequencies (0.5 & 1 kHz) reducing to approximately 4 to 2times at higher carrier frequencies (2 &4 kHz) for FM at both the rates.

A mixed-model ANOVA (for repeated measures) was performed, with carrier frequency (4 levels) and modulation rate (2 levels) as within-subject factors and group (2 levels) as a between-subject factor. To make the variation more uniform, the analysis was performed on the natural log of the threshold values. This revealed a significant main effect of carrier frequency (F_(3, 86.2)_ = 83.5, p<0.01), modulation rate (F_(1, 43.1)_ = 42.5, p<0.001) and group (F_(1, 43.1)_ = 70.3, p<0.01). There was a significant two way interaction between group and modulation rate (F_(1, 43.1)_ = 81.6, p<0.01) and group and carrier frequency (F_(3, 86.1)_ = 85.7, P<0.001) indicating that the difference in sensitivity, between groups was not same for all the carriers and modulation rates. From Figure 2, it can be discerned that the difference between the two groups was higher at low carrier frequencies than at higher carrier frequencies. Bonferroni's post hoc analysis for two-way interaction (modulation rate×group) revealed a significant difference between normal hearing listeners and AN across all carrier frequencies and modulation rates. In normal hearing listeners, performance worsened significantly with increasing modulation rate from 2 to 10 Hz for carrier frequencies of up to 1 kHz (t>2.4, p<0.02), but improved significantly with increasing modulation rateat 2 and 4 kHz (t>5.2, p<0.001). Whereas, in listeners with AN performance improved with increase in carrier frequency (t>3.2, p<0.02) but there was no significant effect of modulation rate (t<1.8, p>0.05).

To assess whether the hearing loss (at 0.5, 1, 2 & 4 kHz) contributed to poorer performance for listeners with AN, the Pearson product moment correlations were calculated between FMDL and audiometric thresholds at 0.5, 1, 2 & 4 kHz. The correlations were low for both the modulations rates(r<0.3, p>0.05). In contrast, significant correlations were noted between the FMDL and peak sensitivity (0.5<r>0.7, p<0.01).

### Speech Identification Score

**Figure 3:**
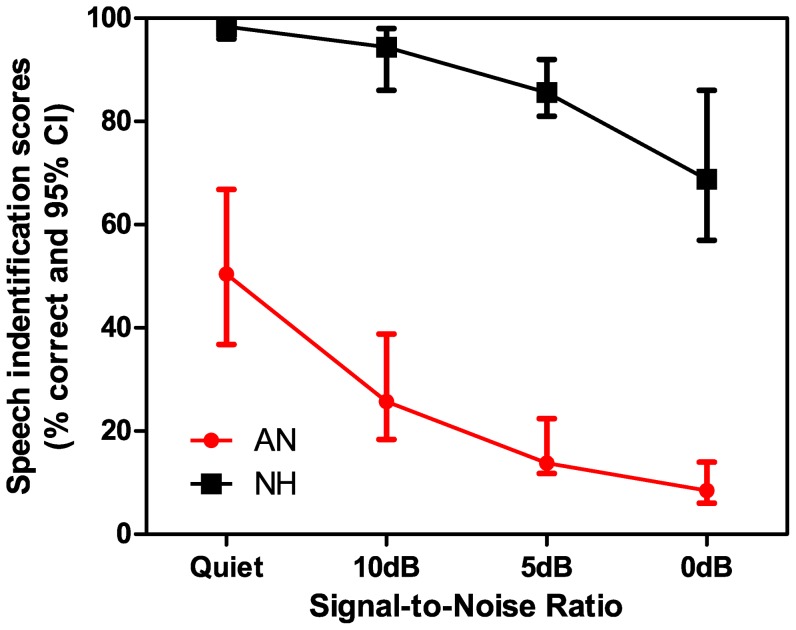
Speech identification scores as a function of signal to noise ratio for listeners with normal hearing (open circle) and those with AN (filled square). The error indicates 95% confidence interval.

Figure 3 presents mean speech identification scores with 95% confidence interval. As the SNR decreased, scores worsened for both the groups but the effect was greater for the group with AN. Further analyses were carried out on ‘rau’ scores (rationalized arcsin transformation), which gives the scores near normal distribution. Kolmogorov-Smirnov's test was performed on the ‘rau’ scores to assess the normality. Results revealed that all but scores at 0 dB SNR showed normal distribution.

A mixed type ANOVA was performed using SNR as within-subject factor (4 levels) and group as between-subject factor (2 levels). This showed a significant main effect of SNR (F_(3, 104)_ = 166.1, p<0.001) and group (F_(1, 43)_ = 194.7, p<0.001). The interaction was significant (F_(3,104)_ = 8.7, p<0.001), indicating that the change in scores with SNR differed between the groups. Bonferroni's post hoc analysis for two-way interaction (SNR×group) revealed that the mean difference reached significance at all the SNR's between normal listeners and AN. Within normal hearing listeners, Bonferroni's post hoc analysis showed that the mean scores in quiet condition were significantly different from those obtained in 5 dB and 0 dB SNR conditions (p<0.01), but they did not differ significantly from those obtained in 10 dB SNR condition. In listeners with AN, results revealed that the mean scores at different SNR conditions were significantly different from each other (p<0.01), except for the scores at 0 dB SNR condition which didn't differ significantly from 5 dB SNR condition.

#### Relation between speech identification scores and psychoacoutical parameters

A multi-factorial regression model was constructed for the prediction of speech identification scores from peak sensitivity (TMTF in dB), cut-off frequency (fc), average pure-tone threshold (PTA), duration of the condition and FMDL (Threshold of 0.5 kHz) in 21 listeners with AN using a general linear model with a best subsets-factorial regression design (Statistica8,StatSoft,Inc., Tulsa, OK). Among the variables included in the model only peak sensitivity, 2 Hz FM at 0.5 kHz and their interaction reached significance (p<0.01) in explaining the variance in identification scores in quiet and all the SNR conditions. Table 2 provides the overall variance that could be explained by including all components (R^2^) and variance explained by individual components (η^2^). It can be discerned from the table that maximum amount of variance that can be explained is by peak sensitivity and second largest contribution came from FMDL at 2 Hz modulation rate at 0.5 kHz.

**Table 2:** Summary of Multivariate Regression Analysis.

		*Proportion Variation %*			
		Quiet	10 dB SNR	5 dB SNR	0 dB SNR
R^2^		86.0*	84.2*	80.2*	68.2*
η^2^	PK	−80.1	41.3	32.2	25.5
	FT	2.5	23.6	23.5	21.8
	PK×FT	3.4	19.3	24.5	22.3
Standard error		10.1	12.1	10.6	9.6
Coefficient	PK	−5.6*	−3.6*	2.2*	−2.1*
	FT	−51.88	−219*	−163*	−42*
	PK×FT	−12.7	−46.33*	−35.6*	−8.9*
	Constant	−8.7	−18.8*	−21.1*	−21.9*

* p<0.01.

PK: Peak Sensitivity obtained in TMTF.

FT: FMDLs at 0.5 kHz for 2 Hz modulation frequency.

PK×FT: Interaction between PK and FT.

### Characteristics of the 4 participants with normal TMTF

A noteworthy observation in the present study was the performance of four listeners with AN, whose identification scores in quiet were greater than 90% and whose amplitude modulation thresholds were within normal limits. The mean FM detection thresholds of these listeners compared to normal hearing listeners were poorer by a factor of 3 to 4 at low carrier frequencies and similar at high carrier frequencies (4 kHz).

**Figure 4:**
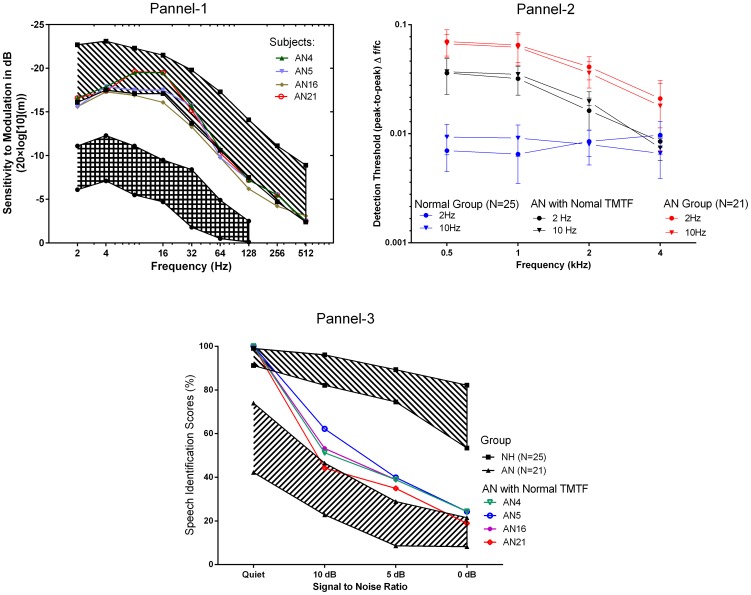
Comparison of data of four exceptional listeners with AN who had normal temporal modulation transfer function (TMTF) and poor perception of speech in noise with the remaining listeners with AN and those with normal hearing. Panel 1: Sensitivity to modulation as a function of modulation frequency. A shaded area with single hatched line for listeners with normal hearing (NH) and double hatched line for listeners with auditory neuropathy (AN). The shaded area indicates 95% confidence interval. Individual data from each of the four listeners with AN is shown with separate symbols. Panel 2: Frequency modulation detection threshold (FMDL)for 2 Hz and 10 Hz expressed as peak-to-peak deviation divided by center frequency and plotted as a function of center frequency. The mean data of the four exceptional listeners with AN is shown separately symbol. Panel 3: Speech perception scores as a function of signal to noise ratio. The shaded area indicates 95% confidence interval. Individual data from each of the four listeners with AN is shown with separate symbols.

Speech identification scores for the four listeners with AN decreased by 30% when the SNR was 10 dB while it decreased by 50% when the SNR was 5 dB compared to quiet condition. With the 0 dB SNR, the scores were 80% lower than those observed in quiet. Figure 4 compares the AM detection thresholds (Panel I), FMDLs (Panel II) and speech identification scores (Panel III) of these four listeners with those of the remaining listeners with AN (21 listeners) and normal hearing listeners.

## Discussion

The present study aims to examine the relation between speech perception scores in quiet and noise with temporal processing ability in listeners with AN. Results of the experiment-1 showed that listeners with AN are impaired in using both temporal envelope (amplitude variation) and temporal fine structure cues. Further, it was also noted that the degree of impairment in processing temporal envelope cues is variable, that is some listeners with AN had no impairment whereas others were unable to use the temporal envelope cues. But majority of the listeners with AN were unable to temporal fine structure.

Results of experiment-2 showed that speech perception scores of listeners with AN are variable ranging from 0% to100%. Adding background noise caused detrimental effects on speech perception scores. Multi-factorial regression analysis showed that impaired ability to follow amplitude variations accounts majorly for impaired speech perception in quiet, whereas speech perception difficulties in noise were accounted by impaired ability to follow amplitude and frequency variations in speech signal. These results are in agreement with our hypothesis that possible reasons for extreme difficulty in understanding speech in the presence of noise by listeners with AN may be associated with impaired processing of both temporal envelope and TFS. These results seem to run in agreement with previous investigations [3], [4], [8].

### Temporal Modulation Transfer Function

To investigate the temporal envelope processing ability in listeners with AN, TMTF was obtained and the TMTF was modeled as a first order low-pass filter. Listeners with normal hearing were more sensitive to lower modulation frequencies (−21 dB at 4 Hz) as compared to higher modulation frequencies (−6 dB at 512 Hz), in turn demonstrating a low pass function. In contrast, majority of the listeners with AN showed higher amplitude modulation threshold to both lower and higher modulation frequencies. The average peak sensitivity obtained at low modulation frequencies for listeners with AN was −9.7 dB and cutoff frequency of the TMTF was 45.2 Hz. Compared to normal hearing listeners, the average peak sensitivity and the cutoff frequency of the TMTF were 12 dB higher and 20 Hz lesser for listeners with AN respectively. Similar differences have been reported in the earlier studies [4], [10].

In the present study, the average peak sensitivity was abnormally high in many listeners with AN with a high degree of variability. Seven out of 21 listeners with AN showed average peak sensitivity in the range of −16 to −12 dB, other seven showed between −12 to −8 dB and rest of the participants showed >−8 dB. In addition to the variability in the peak sensitivity, the shape of the TMTF was atypical in 11 listeners with AN. Among them, seven had flat configuration and the remaining four had band pass shape. A flat configuration was noticed for listeners with average peak sensitivity −8 dB and all of these listeners had very poor speech identification scores. Similar pattern of TMTF was reported by Zeng et al. [4] for 3 listeners whose thresholds were above −6 dB.

**Figure 5:**
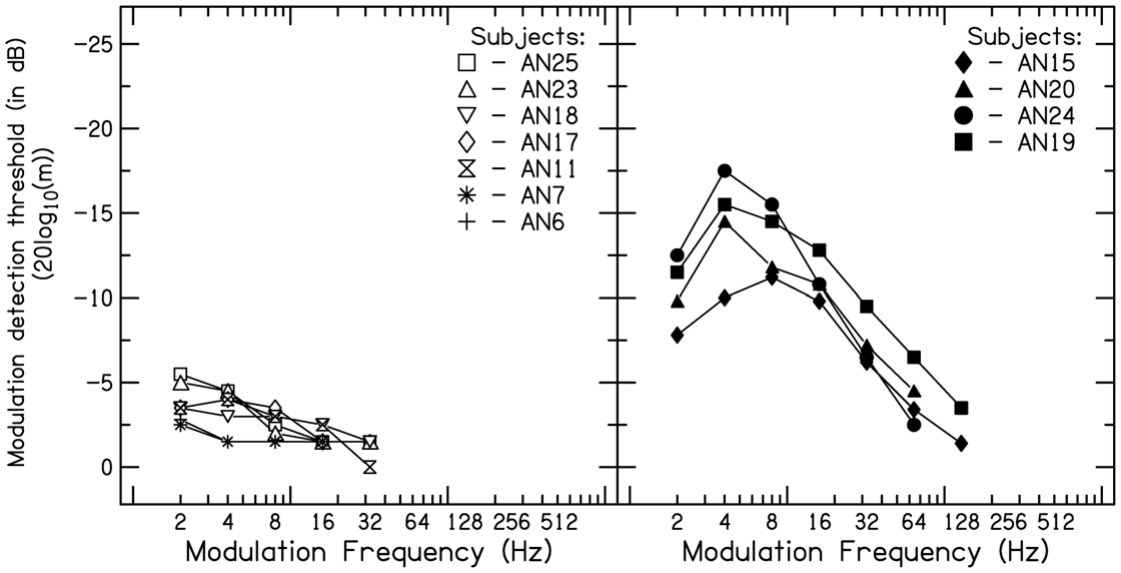
Atypical temporal modulation transfer function (TMTF) pattern measured in listeners with AN. Left panel shows flat pattern and right panel shows band pass pattern. In each panel, the ordinate is the amplitude modulation detection threshold, expressed in decibels as 20 log m and abscissa is modulation frequency (Hz).

The band pass shape of the TMTF noticed in the other four listeners with AN was due to the increased amplitude modulation detection thresholds at 2 Hz. One possible explanation for the poor detection of 2 Hz amplitude modulation by few listeners with AN might be the impaired intensity resolution. Intensity resolution abilities have not been assessed in the present study. However, literature suggests no evidence of impaired intensity resolution in listeners with AN [10]. The atypical patterns of TMTF observed in listeners with AN are presented in Figure 5. Overall, the results clearly demonstrate that poor sensitivity in detecting amplitude modulation is the contributing factor to the reduced *fc*. The reduced sensitivity to detect amplitude modulation and *fc* suggest poor temporal processing in listeners with AN.

Hall and Grose [29] have reported that amplitude modulation detection thresholds reach adult values by 9 years of age. The high amplitude modulation detection threshold observed in listeners with AN in the present study is unlikely to be because of age, since, the age of the listeners considered was 12 years and above. Therefore, age may not have been a contributing factor for the impaired amplitude modulation detection. Instead, the underlying neural pathology may be the causative element for the high amplitude modulation detection thresholds observed in individuals with AN.

The abnormalities in the auditory pathway that lead to the poor temporal processing seen in AN are not clear, as it is difficult to determine the exact mechanism by which temporal cues are disrupted in the affected listeners. Results of electrophysiological tests conducted on listeners with AN indicate two neuro-physiological manifestations, namely, desynchronized spike discharge and/or reduced spike count [30]. According to the model explained by Zeng et al., [10], these disruptions could result in a time smeared neural representation of the acoustic stimulus. The degree of temporal distortion is determined by the severity of the disruptions [2], [4], [31]. Probably, the smeared neural representation causes difficulty in discriminating small variations in the amplitude of sinusoidal amplitude modulated stimuli from un-modulated stimuli. This may be one of the reasons for the variability noted in the amplitude modulation detection threshold across different listeners. However, the impaired ability to accurately encode even low frequency (<16 Hz) amplitude modulation, which was observed in some listeners, point to neural disruption of the order of tens of milliseconds and may suggest a different pathological mechanism.

### Frequency modulation detection

For listeners with normal hearing sensitivity, the FMDLs for low carrier frequencies were lower for 2 Hz modulation rate than 10 Hz rate, while for higher carrier frequencies, the reverse was true. The thresholds in the present study, were similar to those reported in the earlier investigations [32], [33], in the way that they varied with frequency, but the magnitude of FMDL was 1.4 times higher. The lower thresholds reported in the previous studies may be due to the practice effect.

**Figure 6:**
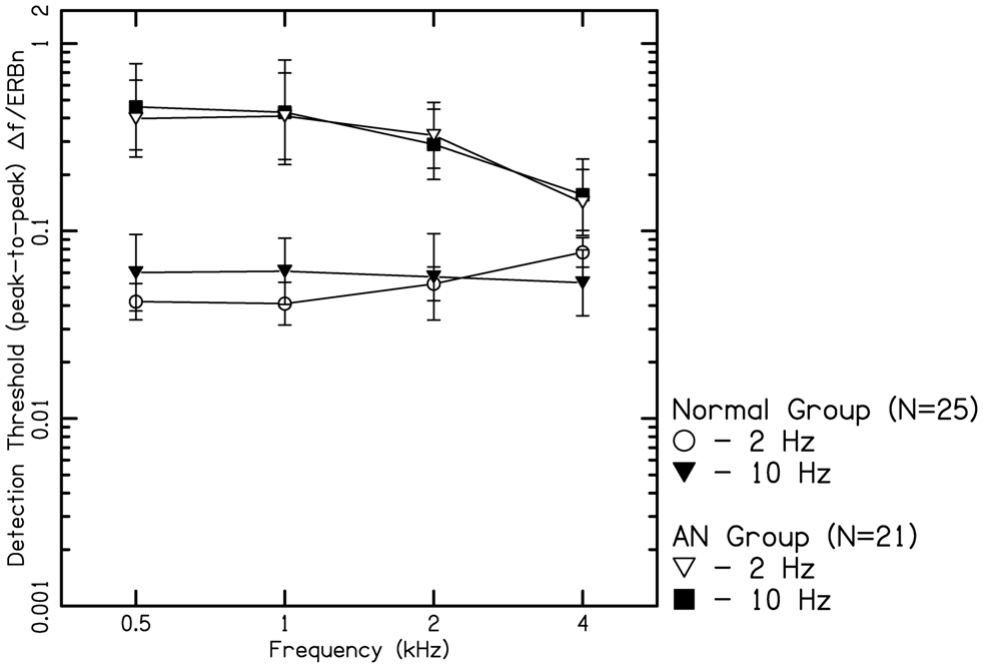
Frequency modulation detection threshold (FMDL) for 2 Hz and 10 Hz modulation frequencies as function of carrier frequencies (0.5, 1, 2, and 4 kHz) expressed as ΔF divided by equivalent rectangular bandwidth (ERB). ΔF/ERB values were grossly abnormal at lower frequencies and lean towards normal function at higher frequencies.

According to Zwicker's model [34], the frequency difference limen (ΔF) divided by the bandwidth of the auditory filter should be a constant. Figure 6 shows mean FMDLs for listeners with normal hearing expressed as a proportion of the equivalent rectangular bandwidth (ERB) of the auditory filter, for which the ERBn values were derived from the equation given by Glasberg & Moore [35]. The ratio FMDLs/ERBn was nearly constant across frequency for the 10-Hz modulation rate. At this rate, the proportions vary by only a factor of 1.2 over the range of 0. 5 to 4 kHz. Thus, the FMDLs at the highest modulation rate (10 Hz) are consistent with the excitation-pattern models. FM at very low modulation rate for low carrier frequencies is detected by virtue of the changes in phase locking to the carrier that occur over time [28]. For the lower modulation rate (2 Hz), the ratios tend to increase at higher carrier frequencies, suggesting that excitation-pattern models do not adequately account for performance over the whole frequency range tested.

From the aforementioned studies, it is noted that detection of FMDLs depends on either the excitation pattern cues or the temporal fine structure processing. Moore and Sek [28] have argued that, for normal hearing listeners, FM at 2 Hz modulation rate is detected via the use of TFS rather than via changes in the excitation pattern, because the former is slightly more effective. However, if the use of TFS information is impaired, listeners can still use excitation-pattern cues. In the present study, listeners with AN performed much more poorly than normal hearing listeners for both modulation rates across all the carrier frequencies. Precisely, it was noticed that the FMDLs for listeners with AN were 10 times higher at low carrier frequencies and 2 to 3 times higher at high carrier frequencies at both the modulation rates than that of listeners with normal hearing. This indicates that in addition to problems with TFS processing, these individuals may have difficulty in using excitation pattern cues as well. Similar results were noted by Rance [3] in children and Zeng et al., [10] in adults with AN for continuous tones. In addition, FMDLs for listeners with AN were twice as high than those reported in cochlear hearing loss by earlier investigators [20]–[22].

Two mechanisms that could disrupt the use of excitation pattern cues are the bandwidth of the auditory filter and the ability to detect the amplitude fluctuations in the low frequency side of the auditory filter [3], [33]. In case of the listeners with AN, it was reported by the previous investigators that the auditory filters were normal [3], [36]. Zwicker [34] stated that the excitation pattern changes that occur as a result of frequency modulation are represented as level variations (amplitude modulations) within each auditory filter. It was hypothesized by Rance et al. [3] that impairment in detection of amplitude modulation will in turn lead to impaired frequency modulation detection. Accordingly, the same was observed in the present study, wherein, it was noted that those listeners who showed poorer amplitude modulation detection also had poorer frequency modulation detection.

However, there were four exceptional listeners with AN, who had TMTF thresholds within normal limits (see Figure 4) but had FMDLs thresholds three times higher than normal hearing listeners at low frequencies, however at high frequencies, FMDLs were within normal limits. These results are ambiguous in light of the reported findings that the auditory filters at low frequencies are normal [3], [36] and as observed in the current study their ability to follow amplitude modulation being within normal limits. Similar results were reported by Rance et al, [3] in children with AN. they noted that at the low carrier frequencies (i.e 500 Hz), the mean FMDLs were twice higher than those observed in children with normal hearing. Based on the above assumptions FMDLs obtained for both FM rates in listeners with AN should have been similar to FMDLs in normal hearing listeners for 10 Hz of FM. This indicates a significantly abnormal auditory filter at low frequencies.

### Speech Identification Scores in Noise

In normal hearing listeners speech identification scores reduced by 5 to 15% at 5 dB SNR and 15 to 30% at 0 dB SNR compared to quiet condition. These results are in close agreement with those of Liu et al. [37], although the comparison is difficult to make between the two studies. Liu et al. [37] reported that identification scores dropped down to 90–95% at 5 dB SNR and 80% at 0 dB SNR. In the present study, scores dropped down to 85% at 5 dB SNR and 70–75% at 0 dB SNR. The discrepancy in the scores obtained in the two studies may be attributed to the test materials used. Though, speech spectrum shaped noise was used in both the studies at different SNRs, the speech material used was bi-syllabic words in the present study whereas the material was sentence list in Liu et al. [41]. These results particularly demonstrate that normal hearing listeners can maintain performance in the presence of competing background noise.

Similar to what has been described in the literature, in the present study, speech identification scores in listeners with AN varied considerably from 0% to 85% in quiet. And the presence of competing background noise reduced the identification scores more dramatically (30% at 10 dB SNR) compared to normal hearing listeners. Also, among the listeners with AN, noise had a more detrimental effect on those who had poor speech identification scores in quiet than the others. Similar results have been reported in adults with AN [6], [12] and in children with AN [38]. These results are consistent with the subjective complaints of individuals with AN. Nonetheless, it was noted that those listeners with AN who had good identification scores in quiet also had steep reduction in the scores (from 100% in quite to 50% in 10 dB SNR) by the addition of noise. The exact mechanism underlining the detrimental effect of noise in individuals with AN is unclear.

A regression and correlation analysis showed that in quiet condition, audibility is not a factor affecting speech identification scores. A major factor that affects identifications scores is the amplitude modulation detection ability. Studies in normal hearing listeners have shown that, when amplitude modulation in the speech signal was reduced by filtering, it caused profound difficulty in understanding speech. Acoustical analysis on these samples showed that, reducing the amplitude modulations of the speech signal affected the segmental cues and salient cues for consonant identification, by blurring/smearing the consonant-vowel distinction [16]. In addition, it was observed from the data of the current study, that seven of the 21 listeners with AN showed mildly impaired modulation detection ability, and all of these subjects demonstrated good open-set speech identification scores (≥60%). In the other seven listeners, the ability to perceive amplitude fluctuations (even at relatively slow modulation rates <16 Hz) was significantly reduced and open-set speech identification scores were below chance level. The significant variability in identification scores noticed in listeners with AN may be due to severity of temporal disruption.

Results of the regression analysis demonstrate that impaired gross and fine temporal processing may account for the significant difficulty in noise. The listeners with AN have difficulty in extracting envelope from speech signal even in quiet condition. Addition of noise to the speech signal reduces the modulation depth and adds spurious modulations [13], [14], in turn exaggerating the problem faced by the listeners with AN. This explanation would explicate the severe degradation seen in speech intelligibility in listeners with AN with moderate and severe impairment in amplitude modulation detection in the presence of background noise

In the present study, identification scores of the four exceptional listeners with AN reduced by 35 to 40% in presence of noise. These scores were similar to the scores obtained by earlier investigators in listeners with normal hearing for speech signals without fine structure information [14], [18]. These results suggest that probably the listeners with AN who obtained 85% to 100% scores in quiet were able to get envelope cues but were unable to extract the fine structure cues. This has been psycho-acoustically supported by the present as well as the previous studies [10] which have stated that listeners with AN have significant impairment in processing fine structure information. It is possible that these listeners were unable to extract the fine structure cues from the speech signal due to the disrupted phase locking ability in them [20] and hence had acute difficulty in understanding speech in adverse noise conditions.

## Conclusions

Consistent with previous studies, the results of the present study demonstrate that listeners with AN have significant difficulty in processing temporal information. Difficulties experienced in understanding speech in quiet have been attributed to impaired ability to process temporal envelope cues. In the presence of noise it may be due to impaired ability to extract temporal envelope and fine structure cues from the speech signal. Future studies are needed in this direction to provide a clear understanding of the contributions provided by the temporal envelope and fine structure cues for speech understanding in these listeners.
